# Supplementary material for: Smc5/6’s multifaceted DNA binding capacities stabilize branched DNA structures
Source: Nat Commun. 2022 Nov 23;13:7179. doi: 10.1038/s41467-022-34928-9 (PMC9684126; doi:10.1038/s41467-022-34928-9)
Supplement: Supplementary file 1 — Supplementary Information [file 41467_2022_34928_MOESM1_ESM.pdf]

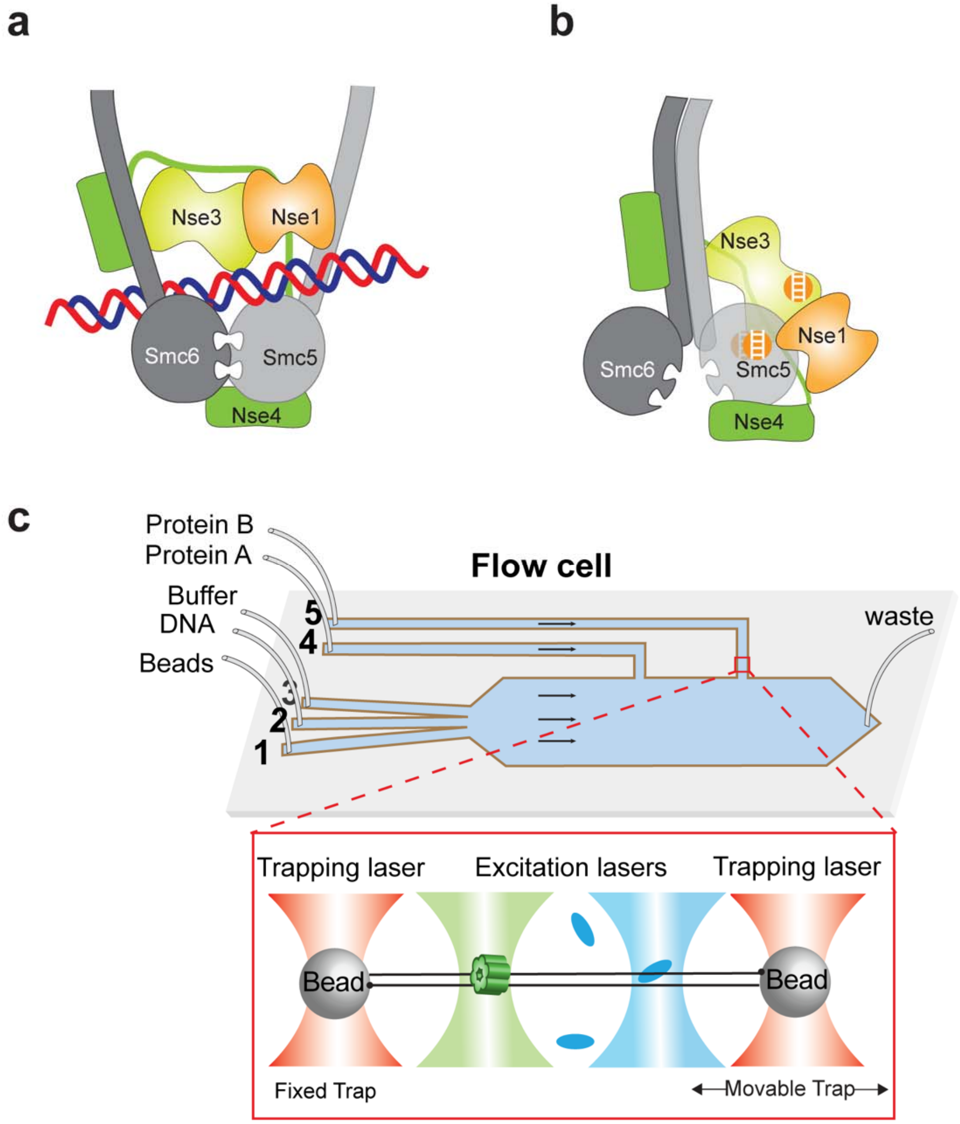

Supplementary Figure 1. Schematics of Smc5/6 structural configurations and single-molecule experimental setup

(**a**) Cartoon of an ATP-bound Smc5/6 complex that forms a clamp to enclose a DNA duplex.
(**b**) Cartoon of an apo-form of the Smc5/6 complex without ATP or DNA. The orange symbols indicate regions of Smc5/6 known to bind dsDNA.
(**c**) Schematic of the single-molecule experimental setup. A single DNA tether is formed in channels 1–3 separated by laminar flow containing streptavidin-coated beads, biotinylated DNA, and buffer, respectively. The tether was subsequently moved to channel 4 or 5 for experiments with proteins of interest. The illustration in the zoom-in box is not drawn to scale.

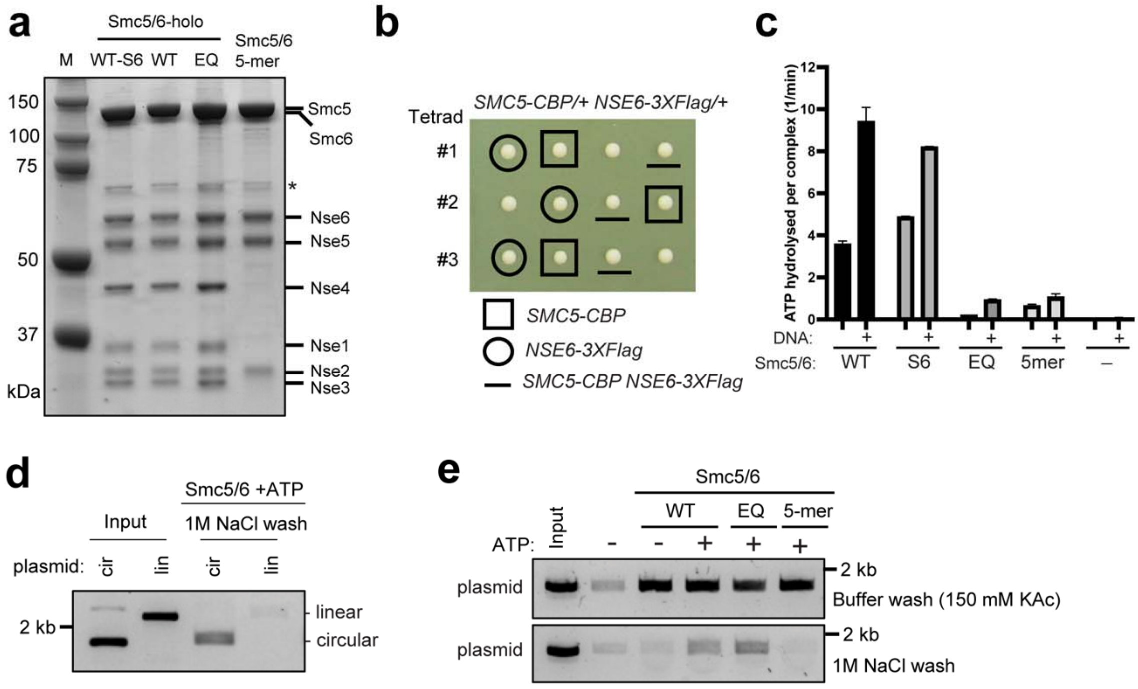

Supplementary Figure 2. Protein purification and bulk biochemical assays

(**a**) A gel picture of purified Smc5/6 complexes, including the wild-type holo-Smc5/6 complex (WT), the wild-type holo-Smc5/6 complex containing a S6 tag on the Smc5 subunit (WT-S6), the ATPase mutant form of the holo-Smc5/6 complex (Smc5-E1015Q, Smc6-E1048Q) and the Smc5/6 complex lacking the Nse1-3-4 subcomplex (Smc5/6 5-mer). A 10% NuPAGE Bis-Tris gel was run in MOPS SDS running buffer and stained using Coomassie blue. The position of each subunit is marked; * marks an unspecific band.

(**b**) Diploid cells containing a copy of Smc5-CBP and a copy of Nse6-Flag at their own chromosomal loci were examined by tetrad analyses. Spore clones containing either tagged allele or both alleles were identified by genotyping the spore clones in 12 tetrads, three of which are shown (#1—3). In all cases, the growth of spore clones of indicated genotype was similar to that of the untagged sibling spore clones without the tag.

(**c**) Examination of the ATPase activities of different forms of Smc5/6 complexes in the absence or presence of a 72-bp dsDNA.

(**d**) An agarose gel showing the amount of DNA recovery when Smc5/6 and ATP were incubated with either a circular plasmid (cir) or a linearized plasmid (lin) and then washed with a high-salt buffer. The Smc5/6 holo-complex can topologically trap circular plasmid but not linearized dsDNA in the presence of ATP.

(**e**) Differential abilities of Smc5/6 complexes (marked as in panel A) to topologically trap circular plasmid. Elutes from both buffer wash and high-salt wash were examined by agarose gel analyses. Raw scans for panels A, D, and E are provided within the Source Data file. These experimental assays for panels A, D, and E were performed twice and yielded reproducible results.

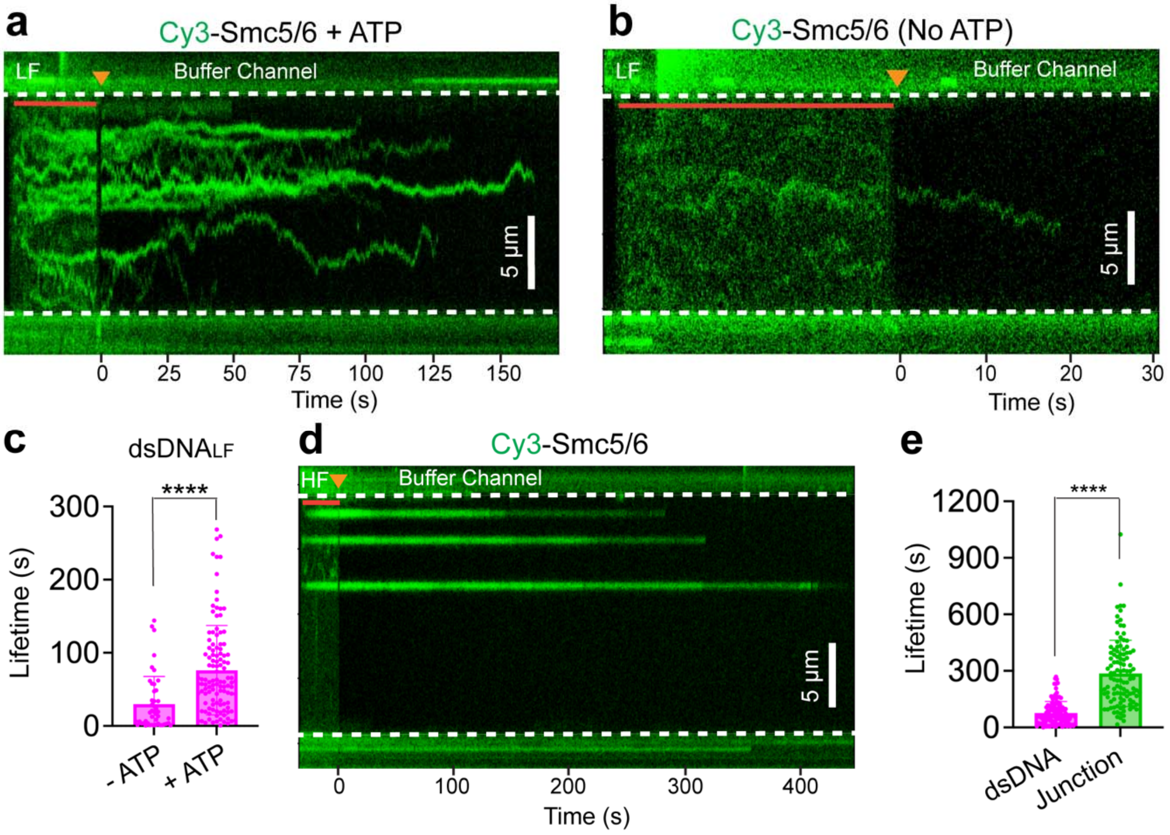

Supplementary Figure 3. Smc5/6 dissociation kinetics on dsDNA and junction DNA
(**a**) Representative kymograph of a λ DNA tether held at 5 pN being incubated with 5 nM Cy3-Smc5/6 (green) and 2 mM ATP for 30 s (red bar) before being moved to a buffer-only channel (orange arrow) where the lifetimes of Smc5/6 trajectories were measured. A total of 21 tethers were evaluated.
(**b**) Same experiment as shown in panel A except that ATP was not included. A total of 30 tethers were evaluated.
(**c**) Lifetimes of Cy3-Smc5/6 trajectories on dsDNA in the buffer-only channel in the absence or presence of ATP.
(**d**) Representative kymograph of a λ DNA tether held at high force being incubated with 5 nM Cy3-Smc5/6 (green) for 30 s (red bar) before being moved to a buffer-only channel (orange arrow). The lifetime of Cy3-Smc5/6 streaks under this condition was likely limited by dye photobleaching (stepwise fluorescence signal decrease was visible after ~200 s).
(**e**) Lifetimes of Cy3-Smc5/6 trajectories on dsDNA versus junction DNA in the presence of 2 mM ATP.
For Panels C and E: Bar heights indicate the group mean and error bars represent standard deviation. *P* values were determined by two-tailed unpaired *t*-tests with Welch's correction (**** $P < 0.0001$). Sample sizes are: dsDNA$_{LF}$ -ATP (n=46); dsDNA$_{LF}$ +ATP (n=116); and Junction$_{HF}$ +ATP (n=109), where n indicates the number of Smc5/6 streaks analyzed. Source data are provided within the Source Data file.

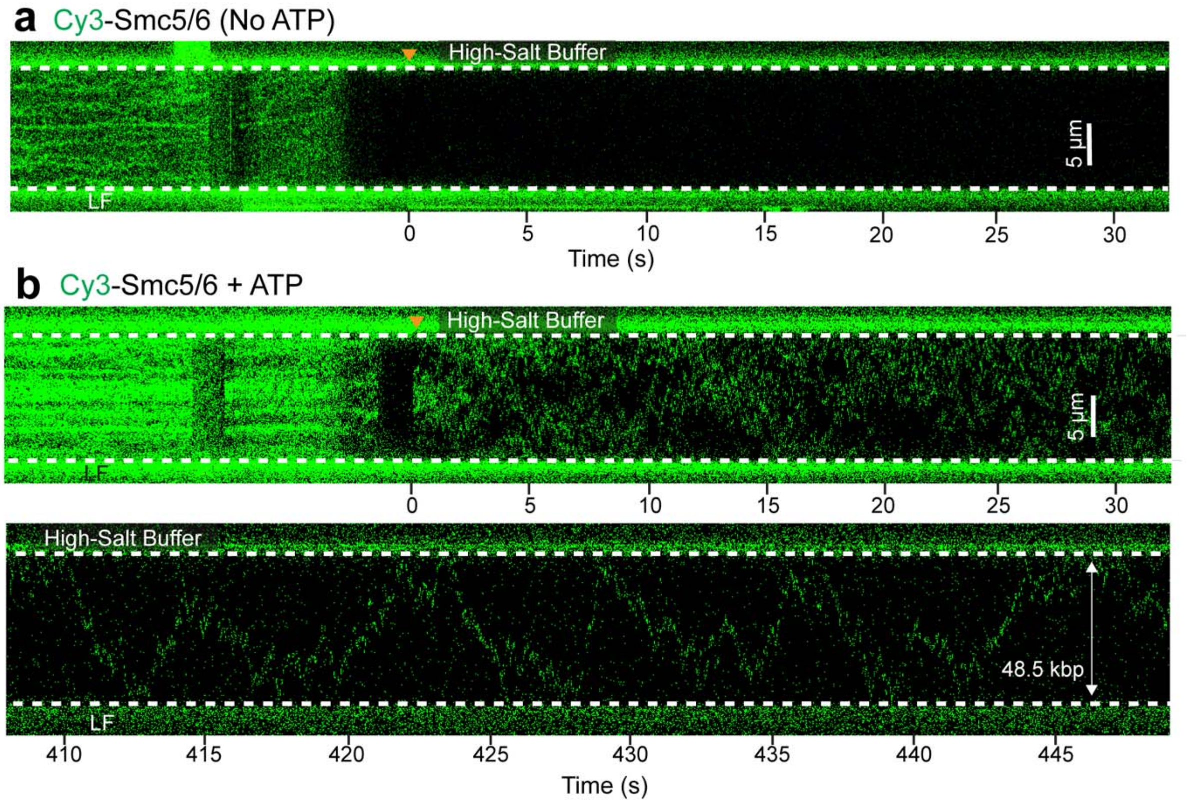

Supplementary Figure 4. High-salt resistance of Smc5/6 association with dsDNA in the presence of ATP

(**a**) Representative kymograph showing dissociation of Cy3-Smc5/6 (green) from dsDNA when the tether was moved to a high-salt channel (500 mM NaCl) in the absence of ATP.

(**b**) Representative kymograph of the same experiment as shown in panel A except that 2 mM ATP was added. (*Top*) Rapid diffusive movements of Smc5/6 complexes were observed in the high-salt channel. (*Bottom*) On the same dsDNA tether shown in the top, Smc5/6 exhibited long-range, rapid diffusion that traversed the entire length of 48.5-kbp lambda dsDNA and persisted after 400 s in the high-salt buffer. Orange arrows indicate when the DNA tether had stopped moving in the high-salt channel and the time ticks indicate the amount of time elapsed in the high-salt buffer.

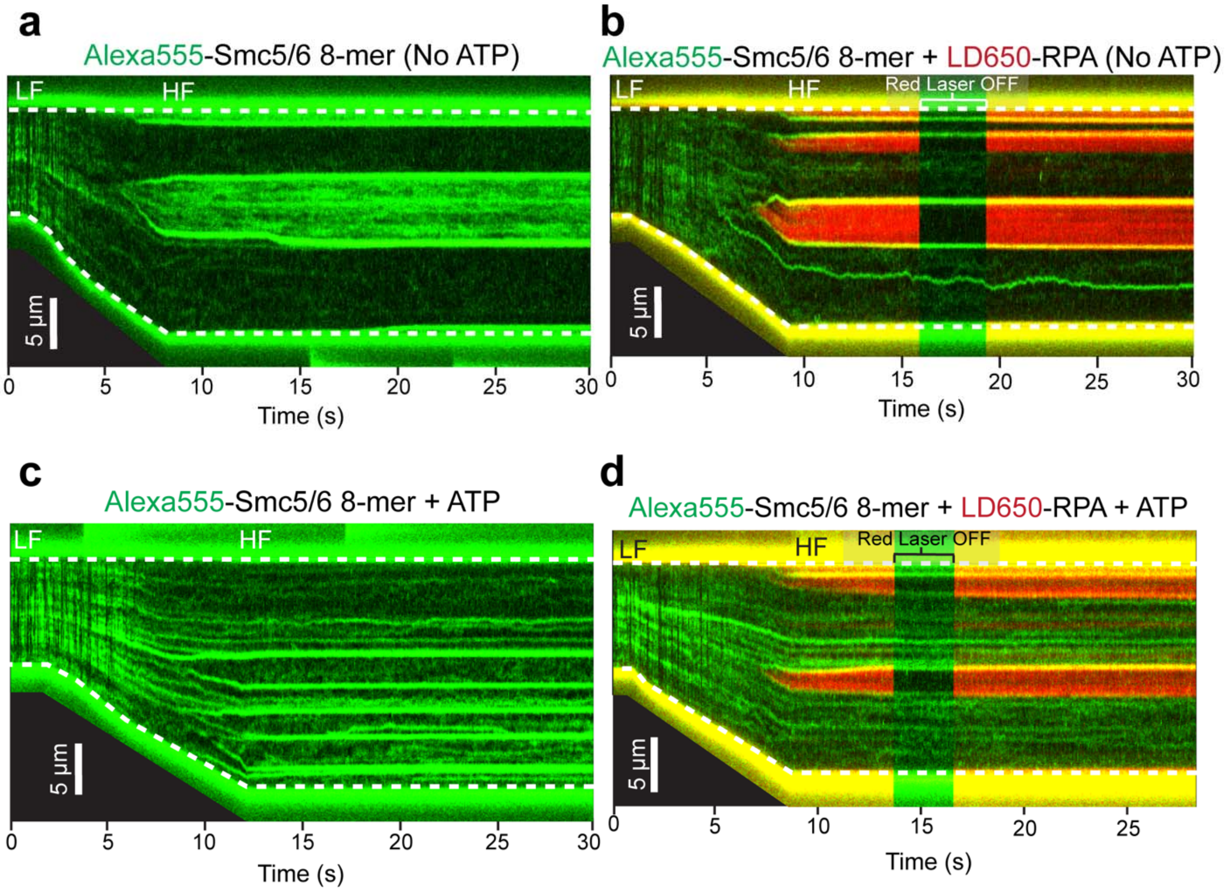

Supplementary Figure 5. Evaluation of the DNA binding behavior of the non-specifically labeled Smc5/6 8-mer complex

Representative kymographs of a λ DNA tether being stretched from LF to HF in the presence of 20 nM Alexa555-Smc5/6 8-mer (green) with or without 10 nM LD650-RPA (red) and/or 2 mM ATP. Each condition has at least five replicates.

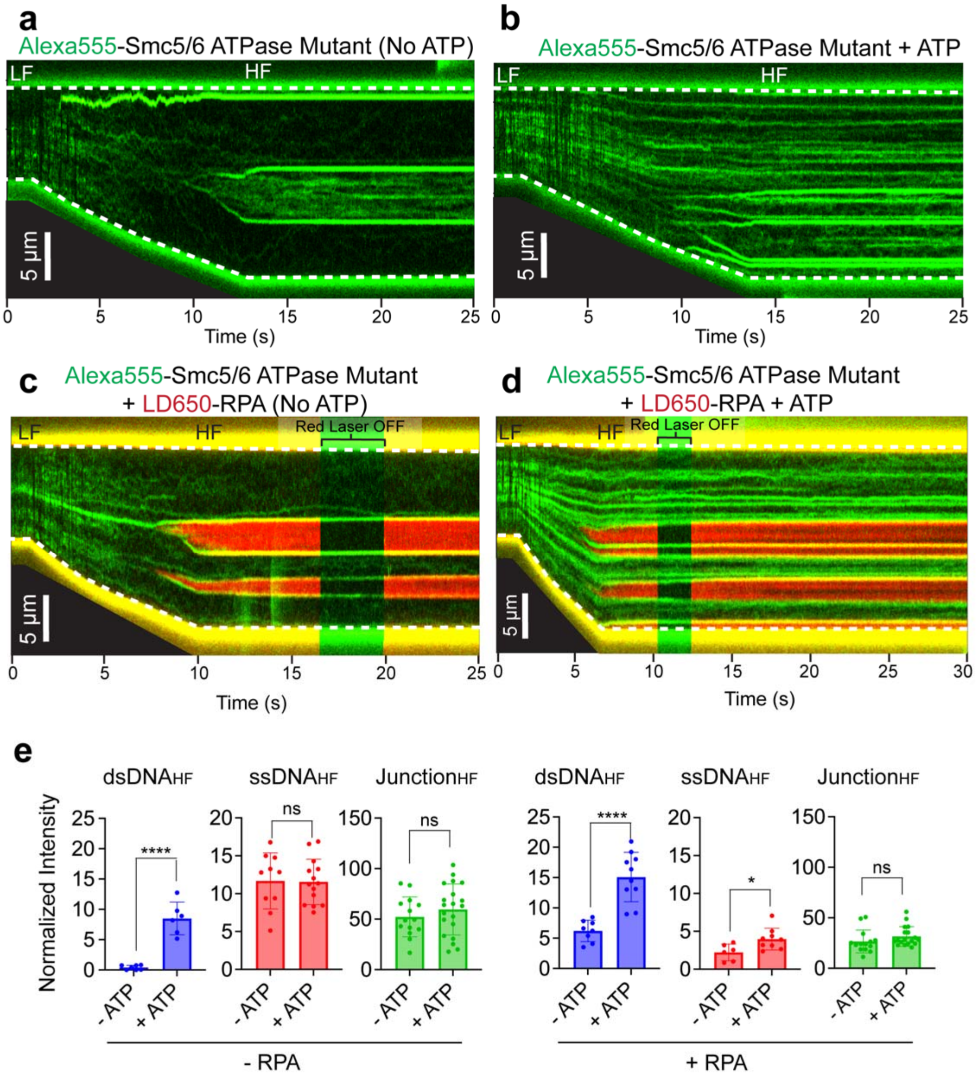

Supplementary Figure 6. Evaluation of the DNA binding behavior of the Smc5/6 ATPase mutant complex

(**a** & **b**) Representative kymographs of a λ DNA tether being stretched from LF to HF in the presence of 20 nM Alexa555-Smc5/6 ATPase mutant (green) without or with 2 mM ATP.

(**c** & **d**) Representative kymographs of a λ DNA tether being stretched from LF to HF in the presence of 20 nM Alexa555-Smc5/6 ATPase mutant (green) and 10 nM LD650-RPA (red) without or with 2 mM ATP.

(**e**) Quantification of the fluorescence signals of Alexa555-Smc5/6 ATPase mutant on dsDNA, ssDNA, and junction DNA (normalized by the number of pixels for each region) at HF under different ATP and RPA conditions. Bar heights indicate the group mean and error bars represent standard deviation. *P* values were determined from two-tailed unpaired *t*-tests (ns, not significant; * $P < 0.05$; **** $P < 0.0001$). $P = 0.9318$ in ssDNA$_{HF}$ -RPA condition; $P = 0.3675$ in Junction$_{HF}$ -RPA condition; $P = 0.0253$ in ssDNA$_{HF}$ +RPA condition; and $P = 0.1888$ in Junction$_{HF}$ +RPA condition. Samples sizes are: dsDNA -RPA -ATP (n=7); dsDNA -RPA +ATP (n=8); ssDNA -RPA -ATP (n=10); ssDNA -RPA +ATP (n=14), Junction -RPA -ATP (n=14); Junction -RPA +ATP (n=20); dsDNA +RPA -ATP (n=8); dsDNA +RPA +ATP (n=10); ssDNA +RPA -ATP (n=6); ssDNA +RPA +ATP (n=9); Junction +RPA -ATP (n=14); and Junction +RPA +ATP (n=18), where n indicates the number of regions analyzed. Source data are provided within the Source Data file.

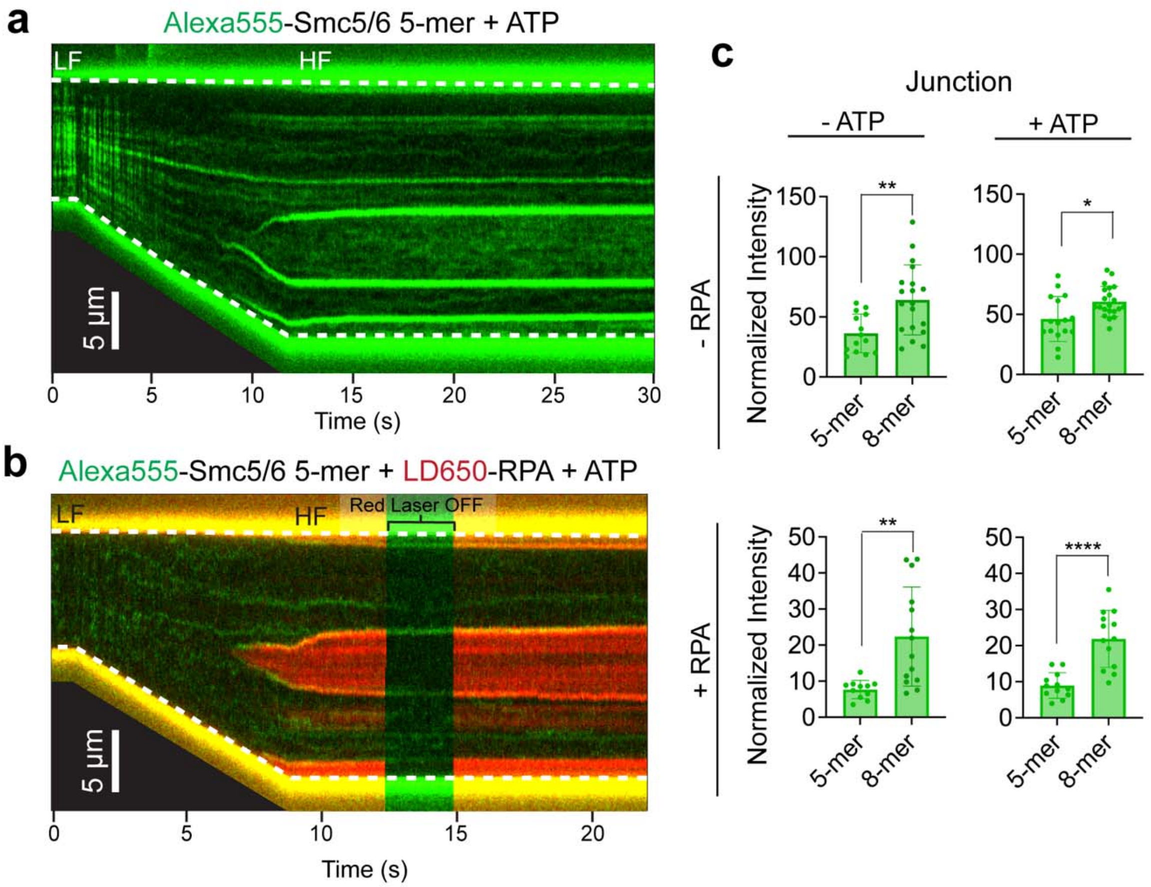

Supplementary Figure 7. Additional evaluation of the DNA binding behavior of the Smc5/6 5-mer complex

(**a**) Representative kymograph of a λ DNA tether being stretched from LF to HF in the presence of 20 nM Alexa555-Smc5/6 5-mer (green) and 2 mM ATP.

(**b**) Representative kymograph of a λ DNA tether being stretched from LF to HF in the presence of 20 nM Alexa555-Smc5/6 5-mer (green), 10 nM LD650-RPA (red), and 2 mM ATP.

(**c**) Comparison of the fluorescence signals at junction DNA between Smc5/6 5-mer and 8-mer complexes under different ATP and RPA conditions indicated in the panels. Bar heights indicate the group mean and error bars represent standard deviation. *P* values were determined from two-tailed unpaired *t*-tests with Welch's correction (* $P < 0.05$; ** $P < 0.01$; **** $P < 0.0001$). $P = 0.0016$ in -RPA -ATP condition; $P = 0.0132$ in -RPA +ATP condition; and $P = 0.0015$ in +RPA -ATP condition. Sample sizes are: 5-mer -RPA -ATP (n=14); 8-mer -RPA -ATP (n=19); 5-mer -RPA +ATP (n=16); 8-mer -RPA +ATP (n=21); 5-mer +RPA -ATP (n=11); 8-mer +RPA -ATP (n=14); 5-mer +RPA +ATP (n=12); and 8-mer +RPA +ATP (n=13), where n indicates the number of junction regions analyzed. Source data are provided within the Source Data file.

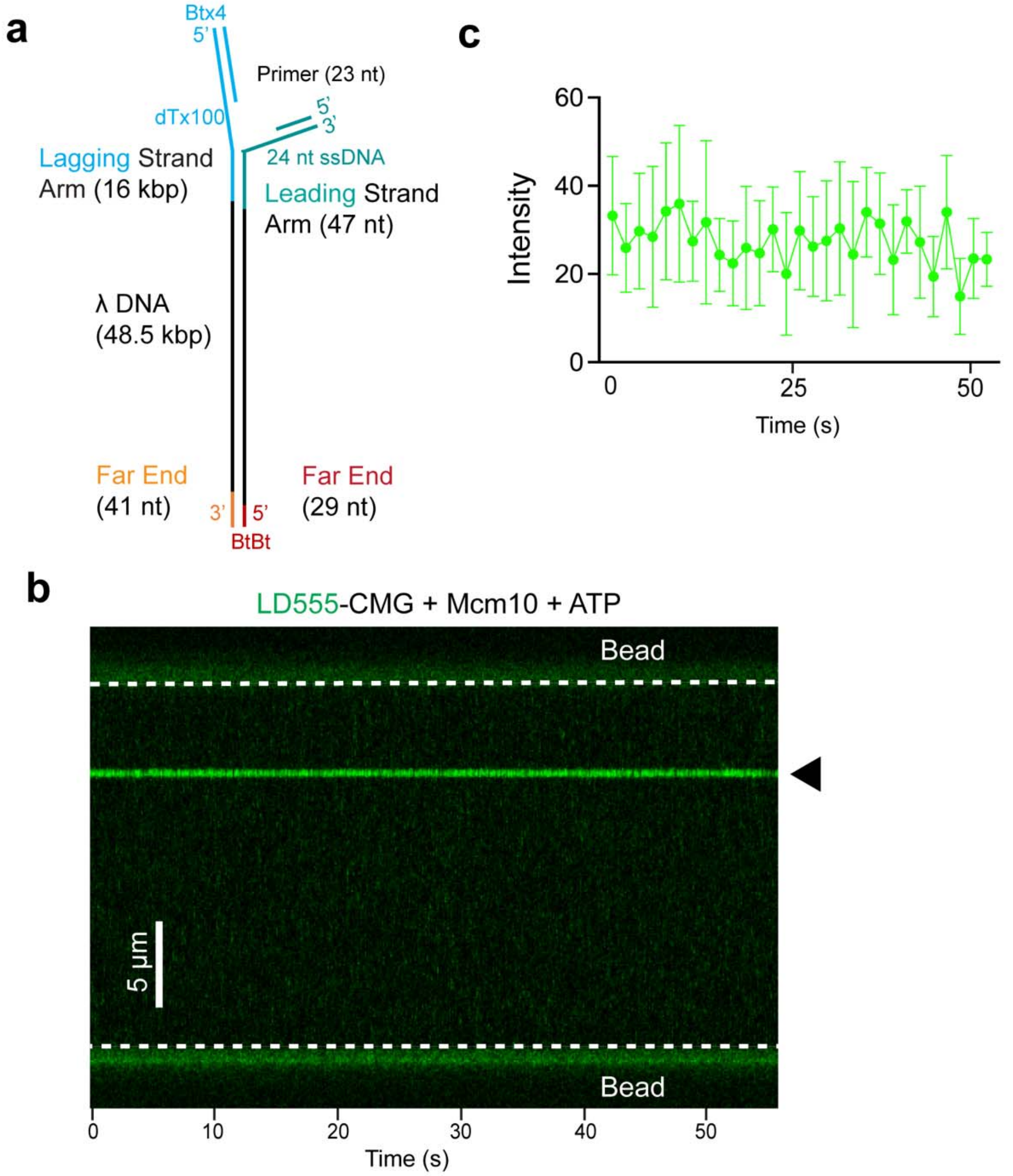

Supplementary Figure 8. DNA construct harboring an intrinsic fork
(**a**) Schematic of the forked DNA substrate.
(**b**) Representative kymograph of the forked substrate held at 5 pN via its parental strand arm and lagging strand arm in the presence of 5 nM LD555-CMG (green), 10 nM Mcm10, and 2 mM ATP showing specific binding of CMG at the intrinsic replication fork.
(**c**) Quantification of the fluorescence signals of the CMG streak (indicated by the black arrow in panel B) over time. Data points indicate the averaged photon count per frame (n=10 frames) at the fork site and error bars represent standard deviation. Source data are provided within the Source Data file.

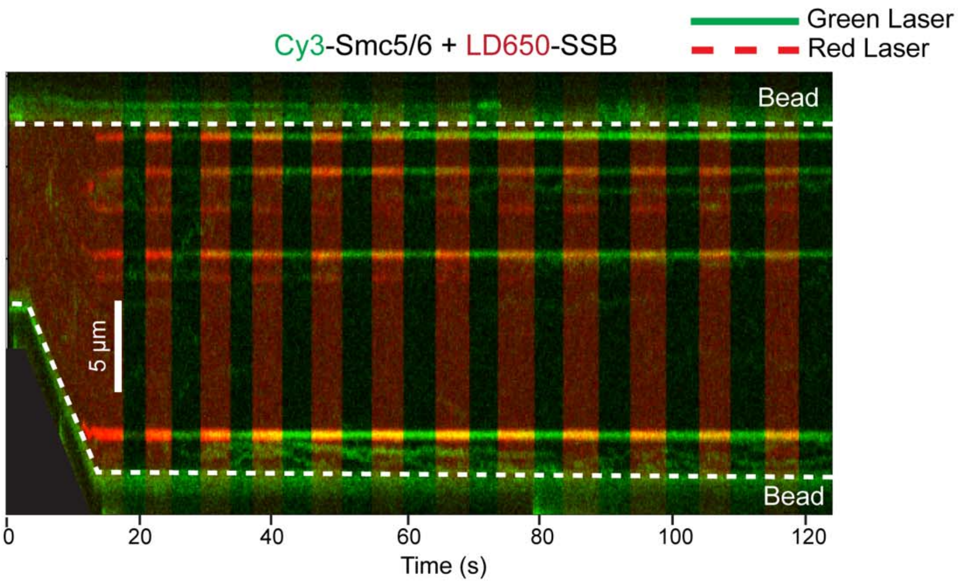

Supplementary Figure 9. Additional example of Smc5/6 assembly on SSB-bound ssDNA Representative kymograph of a λ DNA tether being stretched from LF to HF in the presence of 20 nM Cy3-Smc5/6 (green) and 10 nM LD650-SSB (red). The Smc5/6 and SSB signals were co-localized at junction DNA sites.

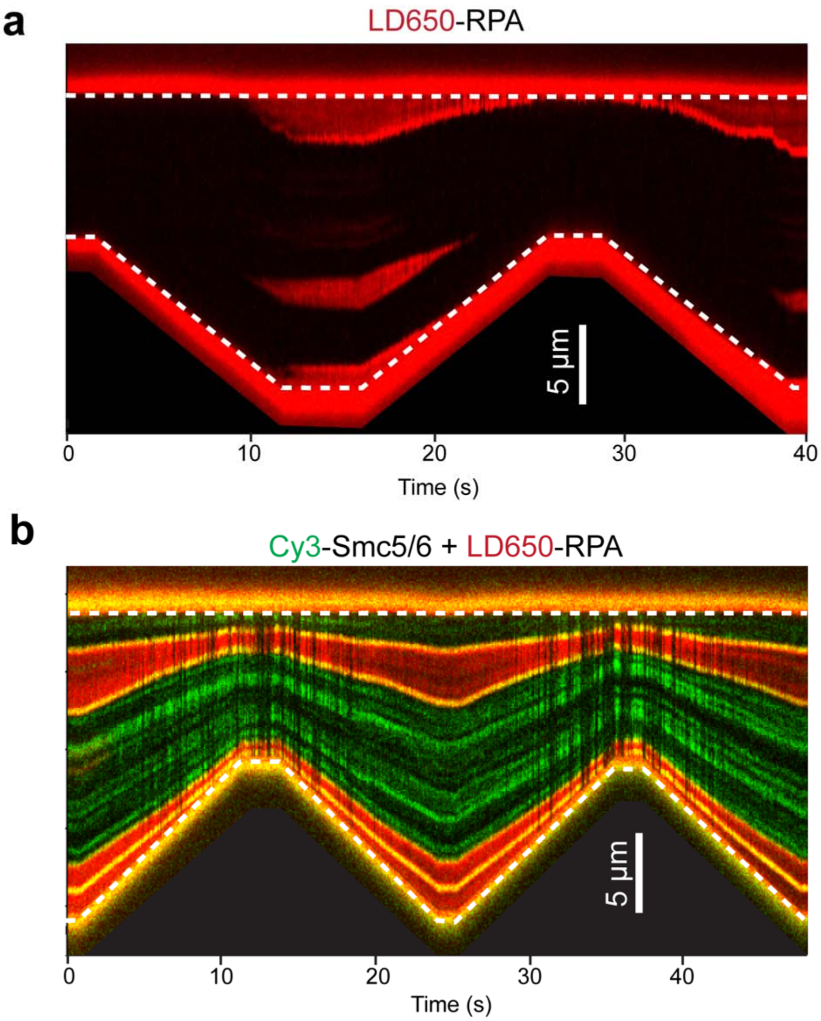

Supplementary Figure 10. Additional examples of Smc5/6 preventing ssDNA annealing at DNA junctions

(**a**) Representative kymograph of a λ DNA tether alternating between LF and HF in the presence of 10 nM LD650-RPA (red). RPA was ejected from the DNA in the transition from HF to LF due to re-annealing of complementary ssDNA strands.

(**b**) Representative kymograph of a λ DNA tether alternating between LF and HF in the presence of 20 nM Cy3-Smc5/6 (green), 10 nM LD650-RPA (red), and 2 mM ATP. The RPA streaks remained on the DNA at LF.
