# Supplementary material for: Smc5/6’s multifaceted DNA binding capacities stabilize branched DNA structures
Source: Nat Commun. 2022 Nov 23;13:7179. doi: 10.1038/s41467-022-34928-9 (PMC9684126; doi:10.1038/s41467-022-34928-9)
Supplement: Supplementary file 3 — Description of Additional Supplementary Files [file 41467_2022_34928_MOESM3_ESM.pdf]

Description of Additional Supplementary Files

File Name: Supplementary Movie 1
Description: Representative continuous 2D scan depicting a λ DNA tether transitioning from LF to HF in the presence of 20 nM Cy3-Smc5/6 (green) and 2 mM ATP.

File Name: Supplementary Movie 2
Description: Representative continuous 2D scan depicting a λ DNA tether transitioning from LF to HF in the presence of 20 nM Alexa555-Smc5/6 ATPase mutant (green), 10 nM LD650-RPA (red), and 2 mM ATP.

File Name: Supplementary Movie 3
Description: Representative continuous 2D scan depicting a λ DNA tether transitioning from LF to HF in the presence of 20 nM Cy3-Smc5/6 (green) and 10 nM LD650-SSB (red).
