# Supplementary material for: Smc5/6’s multifaceted DNA binding capacities stabilize branched DNA structures
Source: Nat Commun. 2022 Nov 23;13:7179. doi: 10.1038/s41467-022-34928-9 (PMC9684126; doi:10.1038/s41467-022-34928-9)
Supplement: Supplementary file 7 — Reporting Summary [file 41467_2022_34928_MOESM7_ESM.pdf]

Corresponding author(s): Shixin Liu, Xiaolan Zhao

Last updated by author(s): Nov 9, 2022

Reporting Summary

Nature Portfolio wishes to improve the reproducibility of the work that we publish. This form provides structure for consistency and transparency in reporting. For further information on Nature Portfolio policies, see our [Editorial Policies](#) and the [Editorial Policy Checklist](#).

Statistics

For all statistical analyses, confirm that the following items are present in the figure legend, table legend, main text, or Methods section.

n/a	Confirmed	
☐	☒	The exact sample size (*n*) for each experimental group/condition, given as a discrete number and unit of measurement
☐	☒	A statement on whether measurements were taken from distinct samples or whether the same sample was measured repeatedly
☐	☒	The statistical test(s) used AND whether they are one- or two-sided<br>*Only common tests should be described solely by name; describe more complex techniques in the Methods section.*
☒	☐	A description of all covariates tested
☐	☒	A description of any assumptions or corrections, such as tests of normality and adjustment for multiple comparisons
☐	☒	A full description of the statistical parameters including central tendency (e.g. means) or other basic estimates (e.g. regression coefficient) AND variation (e.g. standard deviation) or associated estimates of uncertainty (e.g. confidence intervals)
☐	☒	For null hypothesis testing, the test statistic (e.g. *F*, *t*, *r*) with confidence intervals, effect sizes, degrees of freedom and *P* value noted<br>*Give P values as exact values whenever suitable.*
☒	☐	For Bayesian analysis, information on the choice of priors and Markov chain Monte Carlo settings
☒	☐	For hierarchical and complex designs, identification of the appropriate level for tests and full reporting of outcomes
☒	☐	Estimates of effect sizes (e.g. Cohen's *d*, Pearson's *r*), indicating how they were calculated

Our web collection on [statistics for biologists](#) contains articles on many of the points above.

Software and code

Policy information about [availability of computer code](#)

Data collection	Single-molecule experiments were performed on a LUMICKS C-Trap instrument, which combines three-color confocal fluorescence microscopy, dual-trap optical tweezers, and microfluidics technology. Files were exported from the instrument in the .h5 file format for data visualization and analysis.
Data analysis	Force and fluorescence data from the .h5 files generated from C-Trap experiments were analyzed using tools in the lumicks.pylake Python library supplemented with other Python modules (Numpy, Matplotlib, Pandas) in a custom GUI Python script titled "C-Trap .h5 File Visualization GUI" (https://harbor.lumicks.com/single-script/c5b103a4-0804-4b06-95d3-2 0a08d65768f). This script was used to extract confocal images and correlative force measurements. All specified scripts used to run or analyze C-Trap experiments can be accessed on Lumicks Harbor (harbor.lumicks.com).<br><br>The fluorescent quantification analysis to track the intensity of a specific streak involved custom software (https://harbor.lumicks.com/single-script/23f33367-7b02-4762-9457-37348da59194). A series of pixel boxes of dimensions SxlO (i.e. a pixel column of 5 pixels along 10 frames) was created along a fluorescent streak of interest in a kymograph. The summed absolute photon count per frame and the standard deviation of each pixel box could then be plotted on its own or aggregated with calculations from other streaks. Graphs and plots were created with Prism 9 version 9.3.1.<br><br>Supplemental movies were generated by collating continuous 2D scans via ImageJ (Version 1.53).

For manuscripts utilizing custom algorithms or software that are central to the research but not yet described in published literature, software must be made available to editors and reviewers. We strongly encourage code deposition in a community repository (e.g. GitHub). See the Nature Portfolio [guidelines for submitting code & software](#) for further information.

Data

Policy information about availability of data

All manuscripts must include a data availability statement. This statement should provide the following information, where applicable:

- Accession codes, unique identifiers, or web links for publicly available datasets
- A description of any restrictions on data availability
- For clinical datasets or third party data, please ensure that the statement adheres to our policy

Source data are provided with this paper through a Source Data file. Additional data are available from the corresponding authors upon reasonable request.

Human research participants

Policy information about studies involving human research participants and Sex and Gender in Research.

Reporting on sex and gender	N/A
Population characteristics	N/A
Recruitment	N/A
Ethics oversight	N/A

Note that full information on the approval of the study protocol must also be provided in the manuscript.

Field-specific reporting

Please select the one below that is the best fit for your research. If you are not sure, read the appropriate sections before making your selection.

☒ Life sciences ☐ Behavioural & social sciences ☐ Ecological, evolutionary & environmental sciences

For a reference copy of the document with all sections, see nature.com/documents/nr-reporting-summary-flat.pdf

Life sciences study design

All studies must disclose on these points even when the disclosure is negative.

Sample size	For single-molecule experiments, 10-30 kymographs were collected for each condition to establish an observed phenotype, with multiple examples available for a reported finding (can be shared upon request). Three representative kymographs for each condition were selected for quantification purposes. The number of measurements was chosen to ensure sufficient statistical power to distinguish significant differences between our samples. Other biochemical assays were completed twice with reproducible results.
Data exclusions	For single-molecule experiments, tethers that displayed abnormal force-extension behaviors were discarded. Additionally, tethers in which protein aggregates attached to the DNA tether during imaging were also discarded.
Replication	For single-molecule experiments, all representative data shown has been replicated numerous times, in which 10-30 independent/separate DNA tethers demonstrate an observed phenotype. Experimental results and reported/observed phenomena have proven to be reproducible on non-consecutive days (over the course of months for a sample preparation) and across different protein preparations and sample types. For more challenging experiments, we explicitly report the reproducibility rate, such as the percentage of times in which we observed Smc5/6 binding at the intrinsic fork in the replication fork substrate.
Randomization	Randomization was not applicable as there was no grouping in this study.
Blinding	Blinding was not applicable to this study since the results (single-molecule imaging, biochemical measurements) are not subjective examinations by the experimenter. This study does not rely on subjective scoring for its analysis, but rather relies on quantitative data outputs, primarily absolute photon counts.

Reporting for specific materials, systems and methods

We require information from authors about some types of materials, experimental systems and methods used in many studies. Here, indicate whether each material, system or method listed is relevant to your study. If you are not sure if a list item applies to your research, read the appropriate section before selecting a response.

Materials & experimental systems

n/a	Involved in the study
☒	☐ Antibodies
☒	☐ Eukaryotic cell lines
☒	☐ Palaeontology and archaeology
☒	☐ Animals and other organisms
☒	☐ Clinical data
☒	☐ Dual use research of concern

Methods

n/a	Involved in the study
☒	☐ ChIP-seq
☒	☐ Flow cytometry
☒	☐ MRI-based neuroimaging
